# Molecular characterization of *Mycoplasma synoviae* isolated from broiler chickens of West Azarbaijan province by PCR of *vlhA* gene

**Published:** 2016-09-15

**Authors:** Abolfazl Ghaniei

**Affiliations:** *Department of Poultry Diseases, Faculty of Veterinary Medicine, Urmia University, Urmia, Iran.*

**Keywords:** Broilers, Iran, *Mycoplasma synoviae*, *vlhA*

## Abstract

*Mycoplasma synoviae* (MS) is a pathogen responsible for respiratory and locomotor disorders and causes major economic losses in poultry industry. Early and accurate diagnosis of MS infection plays a major role in control of the infection. This study was conducted to characterize Iranian field isolates of MS isolated from broiler chickens of West Azarbaijan province (Northwest of Iran), and differentiate them from vaccine strain MS-H. Two encoding genes, *16S rRNA* and *vlhA* were employed. PCR results using primers related to *16s rRNA* and *vlhA* genes were analyzed and compared. Out of 21 field samples, eight samples (38.0%) were positive using both sets of primers. Amplified products of *vlhA* gene were sequenced for MS strain identification. The results showed that Iranian field isolates of MS had high nucleotide and amino acid similarity. Iranian field isolates were distinct from vaccine strain MS-H. Results presented in this study showed that characterization of field isolates of MS by sequencing of *vlhA* gene and is beneficial for strain typing and differentiating them from vaccine strain. To our knowledge, this is the first study characterizing *vlhA* gene of MS isolates from broiler chickens in the West Azarbaijan province.

## Introduction

*Mycoplasma synoviae* (MS) is an important poultry pathogen; causing infectious synovitis and respiratory disease. Most frequently, respiratory involvement occurs as subclinical upper respiratory disease in which many birds are infected lifelong and become carriers.^1^ It may be transmitted laterally via direct contact and vertically via eggs.^2^ Rapid and accurate identification of MS isolates are of great importance in control of the infection. In this regard, molecular assays such as polymerase chain reaction (PCR) have been applied. Earlier MS specific PCRs were based on the *16S rRNA* gene.^3^^,^^4^ Recently, other genes such as variable lipoprotein hemagglutinin (*vlhA*) are used.

Genome of MS encodes many proteins,^5^ however, only expression of a few of them have been documented.^6^ Hem-agglutinins account among the most important surface proteins involved in colonization and virulence of avian mycoplasmas.^7^ In MS, hemagglutinins are encoded by related sequences of a multigene family referred to as *vlhA* genes.^8^ It was found that *vlhA* antigenic variation was achieved by the *vlhA* gene conversion in which a pseudo-gene sequence replaced the previously expressed sequence in the *vlhA* gene.^9^ Recently, sequence analysis of the single-copy conserved region of the MS* vlhA* gene has been used for investigations of MS strains and epidemiological analyses.^9^^-^^13^ The PCR based mutation detection techniques provide useful and cost-effective alternatives for the direct analysis of genetic variation.^14^

In countries, that poultry flocks are vaccinated with the live MS strain MS-H, such as Iran, gene sequencing and strain typing of MS isolates are of critical importance, due to differentiation between field and vaccine isolates. The main purpose of the present study was to characterize Iranian field isolates of MS and differentiate them from vaccine isolates.

## Materials and Methods

**Samples.** A total number of 21 broiler chicken farms of older than three weeks of age in West Azarbaijan province (Northwest of Iran) were sampled from April 2014 to February 2015. All samples were obtained from un-vaccinated flocks. Four out of 21 samples were taken from apparently healthy flocks and 17 from flocks with respiratory involvement. From each farm, five swab samples obtained from the choanal cleft and trachea and suspended in 1.5 mL of phosphate-buffered saline and considered one sample. 

**DNA extraction.** Each sample (1 mL) was centrifuged for 30 min at 14,000 *g* at 4 ˚C. The supernatant was removed and the contents were dissolved in 25 µL deionized water. Samples were boiled for 10 min and then placed on ice for 10 min. Afterwards, they were centrifuged at 14,000 *g* for 5 min. The supernatant containing DNA was used as template in amplification reaction.^15^

**Polymerase chain reaction.** In this study, a 530 base pair portion of avian mitochondrial DNA was amplified using *12S rRNA* primers to rule out false negative results.^16^

For detection of MS genome in swab samples, 2 sets of primers were used. The first was *16S rRNA* primers. *16S*-F: 5´-GAAGCAAAATAGTGATATCA-3´ and *16S*-R: 5´-GTCGTCT CCGAAGTTAACAA-3´ previously designed by Lauerman *et al.,* ^3^ amplifying a 207 bp region of the *16S rRNA* gene of MS. The PCR reactions were carried out in 25 µL volume of 2.5 µL of 10X PCR buffer, 0.5 µL of dNTP (10 mM), 1 µL of each primer (10 pmol µL^-1^), 0.5 µL of *Taq* DNA polymerase (5U per µL), 0.5 µL of Mgcl2 (50 mM), 17 µL of deionized water and 2 µL of extracted DNA. Thermal condition of amplification included initial denaturation of 95 ˚C for 5 min, followed by 35 cycles of 94 ˚C for 30 sec, 51 ˚C for 30 sec and 72 ˚C for 90 sec. Final extension was done in 72 ˚C for 10 min. 

The second specific MS primers, for amplifying* vlhA *gene, were as the following: *vlhA*-F: 5'- ATTAGCAGCTA GTGCAGTGGCC -3', *vlhA*-R2: 5'- AGTAACCGATCCGCTTAA TGC -3'. The 350-400 bp fragments of MS *vlhA* gene were amplified.^12^ The *vlhA*-PCR mix was performed in a total volume of 25 μL per sample, containing 2.5 μL of 10X PCR buffer, 0.5 μL of 50 mM MgCl2, 0.5 μL of 10 mM dNTPs, 1 μL of each primer, 0.25 μL of *Taq* DNA polymerase (5U per μL). Consequently 17.25 µL of deionized distilled water and 2 μL of extracted DNA as template, were added. The *vlhA*-PCR reaction was conducted in Eppendorff thermal cycler (Eppendorff, Hamburg, Germany) as follows: 5 min at 94 ˚C, followed by 35 cycles of 60 sec at 94 ˚C, 60 sec at 53 ˚C and 1 min at 72 ˚C, with a final extension cycle of 10 min at 72 ˚C. Amplified products were stained using ethidium bromide (0.5 µg per mL) and subjected to agarose gel electrophoresis. 

**Sequencing and data analysis. **Four PCR products of *vlhA* gene of MS isolates (MS01, MS06, MS07, and MS16) were submitted for sequencing to the Bioneer Inc. (Daejeon, South Korea) using *vlhA* primers as the sequencing primers. Nucleotide (nt) and predicted amino acid (aa) sequences data were aligned with clustal W alignment algorithms. The sequence alignments were checked by eye for ambiguities and errors by the examination of chromatograms. Phylogenetic analysis was conducted based on the nt sequences using a distance method and an un-weighted pair group with arithmetic mean and by calculating bootstrap values for 1000 replicates in MEGA software (Version 6.0; Biodesign Institute, Tempe, USA).^17^

## Results

Eight swab samples out of 21 (38.1%) were positive for MS using PCR of both primers (*16S rRNA* and *vlhA*) as diagnostic method for MS. 

Since live MS vaccine was not used in these broiler flocks, amplified products of field strains were compared to vaccine strain (MS-H). Also, some published *vlhA* sequence of field strains were included in this comparison (Table 1). 

**Fig. 1:**
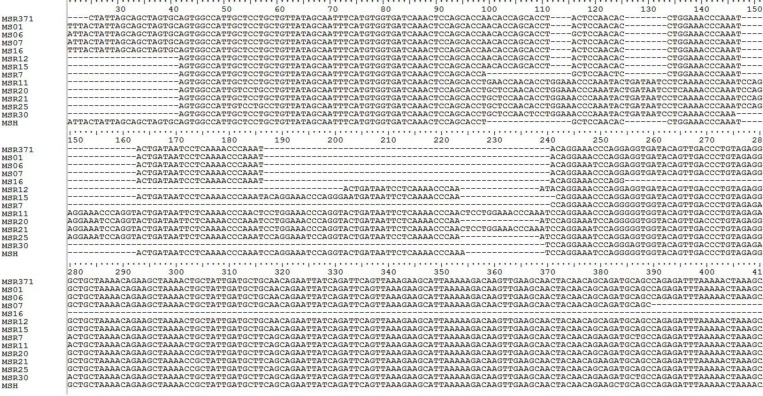
Nucleotide sequence alignment of *vlhA* genes of MS field isolates (MS01, MS06, MS07, and MS16 are isolates of this study; MS371 is from a study by Pourbakhsh *et al.,*^23^; MS12, MS15, MS7, MS11, MS20, MS21, MS25, and MS30 are representatives of eight groups based on Bayatzadeh *et al.,*^24^) and vaccine strain MS-H. Note the 12 additional same nucleotides of MS isolates of the current study, MS371, and MS12, MS15 at the positions 100 to 111, which were not present in vaccine strain MS-H.

Alignment of four field isolates of this study and other Iranian isolates revealed high nucleotide and amino acid similarity (Fig. 1 and Table 2).

**Fig. 2:**
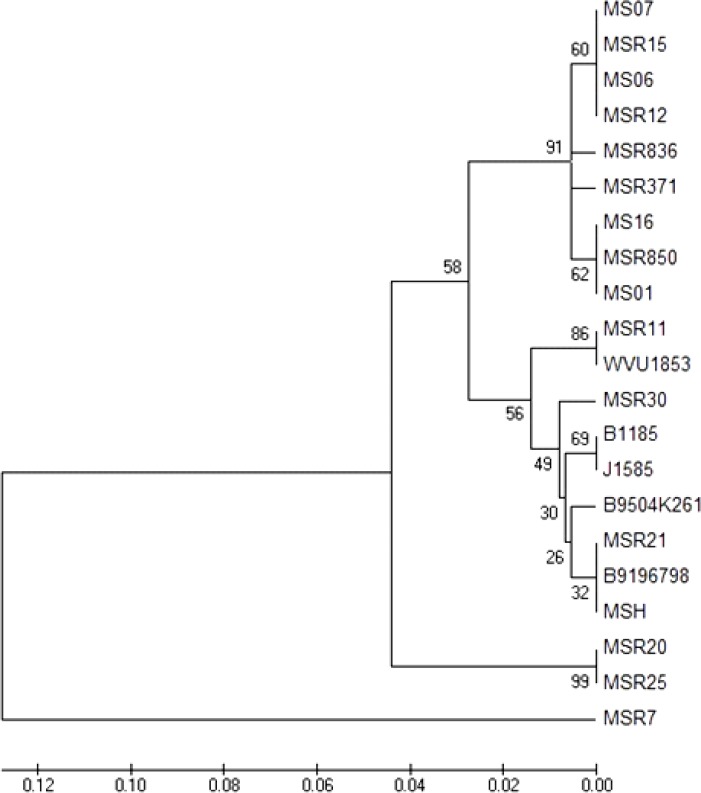
Phylogenetic tree of MS isolates based on the nucleotide sequence of *vlhA* gene. Branched distances correspond to a sequence divergence

Iranian isolates of current study were distinct from vaccine strain MS-H based on sequence alignment similarity and phylogenetic analysis (Fig. 2 and Table 2). 

## Discussion

Most infections of MS occur as subclinical upper respiratory infection. Combination of MS infection with Newcastle disease and infectious bronchitis may lead to air sac disease.^1^ In this study, amplification of *16S rRNA* and *vlhA* genes of MS attempted to demonstrate presence of MS in swab samples taken from broiler flocks with respiratory signs. Two sets of MS specific primers (*16S rRNA* and *vlhA*) were used for comparison and confirmation of the results. Results of the present study showed no difference between PCRs. Ghafouri *et al.* also used two PCRs (*16S rRNA* and *vlhA*) for detection of MS isolates. However, their results showed that results of two sets of primers were not the same.^18^ The MS primers selected from *16S rRNA* gene, published by Lauerman *et al*.^3^ These primers were used by other researchers.^19^^,^^20^ Newer approach to differentiate between MS strains is based on *vlhA* gene. The *vlhA* gene product is an abundant immuno-dominant surface lipoprotein with a conserved and variable region.^9^

**Table 1:** Published MS sequences of *vlhA* used for multiple alignment analysis

**Name**	**Gene bank accession no.**	**Country of origin**
* **MS01**	KT880075	Iran
* **MS06**	KT880076	Iran
* **MS07**	KT880077	Iran
* **MS16**	KT880078	Iran
**MSR836**	JX233544.1	Iran
**MSR371**	JX233546.1	Iran
**MSR850**	JX233549.1	Iran
**MSR-20 ** ^1^	JX960386	Iran
**MSR-25 ** ^2^	JX960390	Iran
**MSR-12 ** ^3^	JX960384	Iran
**MSR-15 ** ^4^	JX960385	Iran
**MSR-30 ** ^5^	JX960392	Iran
**MSR-11 ** ^6^	JX960383	Iran
**MSR-21** ^ 7^	JX960387	Iran
**MSR-7 ** ^8^	JX960381	Iran
**MS-H**	AF464936.1	Australia
**B1185**	FM164346	UK
**B9504K261**	FM164372	Germany
**B9196798**	FM164349	UK
**J1585**	AJ580981	UK
**WVU1853**	AM998371	USA

* indicates MS field isolates of the current study; Superscript numbers indicate group number based on Bayatzadeh *et al.,* classification.^ 24^

**Table 2:** Percentage of nucleotide and amino acid identities for the *vlhA* genes of 19 strains of *Mycoplasma synoviae*^a^. MS01, MS06, MS07, and MS16 are sequences that identified in current study; MSR371 is representative of Iranian field isolates from Pourbakhsh *et al. *study. ^23^

***Strains***	***Similarity***																			
		*1*	*2*	*3*	*4*	*5*	*6*	*7*	*8*	*9*	*10*	*11*	*12*	*13*	*14*	*15*	*16*	*17*	*18*	*19*
**MS01**	1		98	99	100	82	86	78	91	79	99	69	69	98	89	87	66	68	72	73
**MS06**	2	99		98	98	83	87	79	92	78	98	69	69	98	88	88	66	68	72	73
**MS07**	3	99	99		100	77	86	75	87	76	98	64	64	98	86	84	61	63	67	71
**MS16**	4	100	99	100		83	81	84	82	88	100	59	59	100	100	85	63	64	100	84
**B1185**	5	97	97	96	88		82	92	88	93	83	86	85	82	91	82	79	82	74	85
**B9504K261**	6	92	93	91	87	94		88	91	88	86	75	75	85	77	95	71	73	87	79
**B9196798**	7	94	94	92	89	97	98		82	97	76	83	84	77	84	85	78	81	76	90
**J1585**	8	95	95	93	88	98	96	94		82	90	75	75	90	81	94	72	72	85	77
**MS-H**	9	88	88	86	90	96	98	99	95		76	84	83	77	86	84	81	85	75	90
**MSR371**	10	100	99	99	100	97	92	94	95	100		69	72	100	90	88	67	68	79	76
**MSR20 ** ^1^	11	95	96	94	87	96	97	96	96	97	95		99	69	77	73	90	93	67	80
**MSR25 ** ^2^	12	96	96	95	87	96	97	96	96	97	96	100		72	77	74	89	92	68	80
**MSR12 ** ^3^	13	100	99	99	100	97	92	93	94	87	100	95	96		90	88	67	68	79	75
**MSR15 ** ^4^	14	97	96	95	100	95	92	92	96	92	97	93	93	97		79	76	73	67	80
**MSR30 ** ^5^	15	92	92	91	89	94	97	94	97	95	92	96	95	92	93		73	74	90	79
**MSR11 ** ^6^	16	96	96	95	89	91	98	90	98	91	96	94	93	96	84	99		98	70	77
**MSR21 ** ^7^	17	96	96	94	89	90	99	91	96	92	96	95	94	96	84	98	98		67	80
**MSR7 ** ^8^	18	96	95	95	100	97	97	97	97	97	96	98	97	96	95	91	99	98		70
**WVU1853**	19	95	94	94	88	92	97	93	95	95	95	91	92	94	87	95	88	88	97	

a Percentage of amino acid identity is in upper triangle; percentage of nucleotide identity is in lower triangle; Superscript numbers indicate group number based on Bayatzadeh et al. classification.

Recently, sequence analysis of the single-copy conserved region of the MS *vlhA* gene has been used for investigations of MS strains and epidemiological studies.^9^^,^^12^^,^^13^ In countries like Iran that poultry flocks are vaccinated with live MS-H vaccine, differentiating of field and vaccine strains has critical importance. Ghafouri *et al.,*^18^ Ansari *et al.,*^21^ Jamshidi *et al.,*^22^ and Pourbakhsh *et al.,*^23^ used *vlhA* based PCR for differentiation of Iranian field isolates of MS. In order to differentiate field and vaccine strains of MS, Bayatzadeh *et al.* analyzed and sequenced *vlhA* gene of 21 Iranian field isolates. They also used PCR-restriction fragment length polymorphism (RFLP) for characterization of isolates. They stated that DNA sequence analysis and PCR-RFLP were suitable tools for distinction between wild type and vaccine strains of MS.^24^ Amplification of haemagglutinin-encoding *vlhA* gene, sequencing and phylogenetic studies have been reported earlier by researchers to apperceive the relationships between the MS field and MS-H strain.^2^^,^^14^^,^^23^

Broiler flocks of older than 3 weeks old with respiratory involvement were investigated to elucidate role of MS in respiratory complexes. Eight samples (38.1%) out of 21 were positive using both MS specific primers. Four apparently healthy flocks were also included in this survey. Two of them were positive, that emphasize role of MS as subclinical respiratory pathogen. Bayatzadeh *et al.,* analyzed 43 broiler flocks for MS contamination. They noted 55.9% of swab samples were positive by PCR of *16S rRNA*.^25^ In another study, 24 (55.0%) out of 43 samples of suspected flocks of three provinces of Iran were positive by PCR of *vlhA.*^26^ Results of above mentioned studies indicated relatively high prevalence of MS in poultry flocks of Iran. 

Bayatzadeh *et al.,* classified Iranian field isolates of MS to eight groups based on sequence similarity and phylogeny.^24^ Three out of four Iranian strains of current study including MS01, MS06, and MS07 had high sequence similarity with strains of group 3 (MSR12 is representative of group 3). MS16 had high sequence similarity with strains of group 3 and 4 (MSR15 is representative of group 4), (Table 2). Phylogenetic analyses based on nucleotide sequences also showed that Iranian field isolates of the current study clustered together with strains of group 3 and 4 (Fig. 2). It must be noted that nucleotide and amino acid sequence alignments of MS16 and MSR7 (representative of group 8 in Bayatzadeh *et al.*^24^ scheme) were the same (Table 2). However, phylogenetic analysis showed that they were distinct from each other.

Alignment of Iranian field isolates and MS-H showed that these isolates had 12 additional nucleotides, which were absent in MS-H (Fig. 1). Bayatzadeh *et al.* stated that Iranian isolates in groups 3 and 4 had 12 additional identical nucleotides, which were not present in MS-H vaccine strain.^24^ This was consistent with our findings. Based on sequence similarity and phylogeny, isolates of this study belonged to groups 3 and 4. Ogino *et al.,* also noted 12 additional nucleotide in Japanese field isolates, which were not present in MS-H.^27^ They suggested this difference as a method for rapid identification of field and vaccines strains.

Iranian field isolates in the present study had high nucleotide and amino acid similarity (>98.0%). MS01, MS06, MS07, and MS16 had 88.0%, 88.0%, 86.0%, and 90.0% nt identity with MS-H, respectively. At amino acid level, these numbers were somewhat different. Amino acid identity of MS01, MS06, MS07, and MS16 with MS-H were 79.0%, 78.0%, 76.0%, and 88.0%, respectively. Four Iranian MS isolates of this study had G at nucleotide position 106 (Fig. 1). Isolates that were representatives of groups 3 and 4 (i.e. MSR12 and MSR15) according to Bayatzadeh *et al.,* ^24^ scheme, also had G at this position. 

Phylogenetic analysis of the *vlhA* gene of MS strains revealed that Iranian field isolates of current study clustered independently from the isolates of other countries and vaccine strain MS-H. Bayatzadeh *et al.* also cited that MS isolates of Iran are local strains.^24^

This study certified the potential value of strain typing for epidemiological reasons and suggested that phylo-genetic study of *vlhA* genes was essential to understand the true relationships between strains. Such investigations provide researchers with a better knowledge on the distribution, variability, and phylogenetic relationships of different MS isolated in Iran and other parts of the world.
